# Quantifying uncertainty in brain network measures using Bayesian connectomics

**DOI:** 10.3389/fncom.2014.00126

**Published:** 2014-10-08

**Authors:** Ronald J. Janssen, Max Hinne, Tom Heskes, Marcel A. J. van Gerven

**Affiliations:** ^1^Department of Artificial Intelligence, Donders Institute for Brain, Cognition and Behavior, Radboud University NijmegenNijmegen, Netherlands; ^2^Machine Learning Group, Institute for Computing and Information Sciences, Radboud University NijmegenNijmegen, Netherlands

**Keywords:** connectomics, Bayesian inference, diffusion weighted imaging, graph theory

## Abstract

The wiring diagram of the human brain can be described in terms of graph measures that characterize structural regularities. These measures require an estimate of whole-brain structural connectivity for which one may resort to deterministic or thresholded probabilistic streamlining procedures. While these procedures have provided important insights about the characteristics of human brain networks, they ultimately rely on unwarranted assumptions such as those of noise-free data or the use of an arbitrary threshold. Therefore, resulting structural connectivity estimates as well as derived graph measures fail to fully take into account the inherent uncertainty in the structural estimate. In this paper, we illustrate an easy way of obtaining posterior distributions over graph metrics using Bayesian inference. It is shown that this posterior distribution can be used to quantify uncertainty about graph-theoretical measures at the single subject level, thereby providing a more nuanced view of the graph-theoretical properties of human brain connectivity. We refer to this model-based approach to connectivity analysis as Bayesian connectomics.

## 1. Introduction

Connectomics refers to the field of research that aims to unravel the connectivity pattern between distinct neural populations within a nervous system (Sporns et al., 2005; Behrens and Sporns, 2012; Seung, 2012; Sporns, 2012). At the macroscopic scale, connectomics strongly relies on non-invasive mapping of anatomical pathways between brain regions using diffusion weighted imaging (DWI) (Behrens and Sporns, 2012). The resulting structural connectivity estimates have been used to subdivide brain regions into functionally coherent clusters via the notion of connectivity-based parcellation (Beckmann et al., 2009; Cloutman and Ralph, 2012; Mars et al., 2012). Variability in structural connectivity has also been related to individual differences (de Schotten et al., 2011; Catani et al., 2012). Furthermore, changes in structural connectivity have been implicated in several neurodegenerative diseases (Bassett et al., 2008; Riedl and Honey, 2008; Seeley et al., 2009; Lo et al., 2010; Raj et al., 2012). It has become commonplace to summarize structural networks in terms of a wide variety of graph-theoretical measures (Stam and Reijneveld, 2007; Bullmore and Sporns, 2009; Rubinov and Sporns, 2010; van den Heuvel and Sporns, 2011), each reflecting different aspects of network topology.

The quest for the brain's connectome is complicated, however, by the fact that structural connectivity must be inferred from noisy measurements. In case of DWI, these measurements pertain to the anisotropic diffusion of water (Jones et al., 2013). With DWI, one obtains an estimate of water diffusion for each voxel in a number of different directions. This voxel-wise diffusion profile can be modeled as an ellipsoid, which serves to determine the principal diffusion direction in that voxel. By connecting the principal diffusion directions, one can draw a streamline representing a putative axon bundle, connecting two regions in a process known as deterministic tractography (Basser et al., 2000). However, deterministic tractography completely ignores the uncertainty about diffusion direction in individual voxels.

An alternative to deterministic tractography is probabilistic tractography, where streamlines are sampled using a distribution of principal diffusion directions (Behrens et al., 2003). By repeating this process, one constructs distributions of streamlines that reflect the uncertainty in streamlining. An often used heuristic to infer structural connectivity from these distributions is to assume the presence of a connection between brain regions if the number of streamlines connecting those regions survives an (arbitrary) threshold. Hence, in the end there is still only a point estimate of the graph and all graph theoretical measures are, therefore, also limited to point estimates that do not take the stochastic nature of streamlining into account.

Summarizing, current approaches to inferring structural connectivity either assume noise-free data (deterministic streamlining) or use an arbitrary threshold (probabilistic streamlining). Hence, conclusions drawn based on graph-theoretical measures derived from either approach ignore the inherent uncertainty in structural connectivity estimation, possibly leading to erroneous conclusions. To solve this issue, we advocate a fully Bayesian approach to estimating neural connectivity patterns, which we refer to as Bayesian connectomics, or BaCon for short.

## 2. Bayesian connectomics

Bayesian approaches have become increasingly prevalent in neuroscience, providing normative and descriptive models of human cognition (Griffiths et al., 2008; Clark, 2013), as well as a methodologically sound approach to neural data analysis (Penny and Friston, 2006; Friston et al., 2008). In the same vein, Bayesian connectomics makes use of a generative model in order to explain observed data. That is, we propose to infer a probability distribution over brain networks based on (i) our prior knowledge about brain networks and (ii) a forward model, or likelihood function, that explains our measurements.

Several methods have been proposed that we would also consider a BaCon approach. These include the Bayesian approaches to probabilistic streamlining like those proposed in Behrens et al. (2007). These approaches could also be used to propagate uncertainty to graph level analysis by considering one iteration over seed voxels as a sample and draw one streamline per modeled fiber in a voxel. Each of these samples could then be used to construct a graph. Another example would be the tract-level formulation proposed by Jbabdi et al. (2007) and used in the TRACULA framework (Yendiki et al., 2011). These approaches, however, do not lend themselves well to incorporating assumptions at the level of graph structure.

In our recent work, we proposed a model formulated at the network level for structural connectivity (Hinne et al., 2013), allowing us to place a prior on graph structure. Our results demonstrated significantly improved macroscopic structural connectivity estimates using the Bayesian approach, as compared with standard approaches. This previous work focused on the computational details of inferring a distribution over structural networks. In the present work, we use an extension of the original model and show, for the first time, how uncertainty in structural connectivity estimates impacts derived graph-theoretical measures. We proceed by describing the details of this model.

Let structural networks be represented by a symmetrical *K* × *K* adjacency matrix **A**, where *K* is the number of regions (in this work we use the AAL template excluding cerebellum with *K* = 90). Here, *a*_*ij*_ = *a*_*ji*_ = 1 indicates that a direct anatomical pathway exists between brain regions *i* and *j*, and *a*_*ij*_ = *a*_*ji*_ = 0 indicates its absence. The diagonal of this matrix is fixed to 0 to exclude self-loops. We assume that the observed data is given by a *K* × *K* streamline count matrix **N** as generated by a probabilistic tractography algorithm. Under the generative model, the observed data **N** and structural network **A** have a joint probability distribution given by

**Figure 1:**
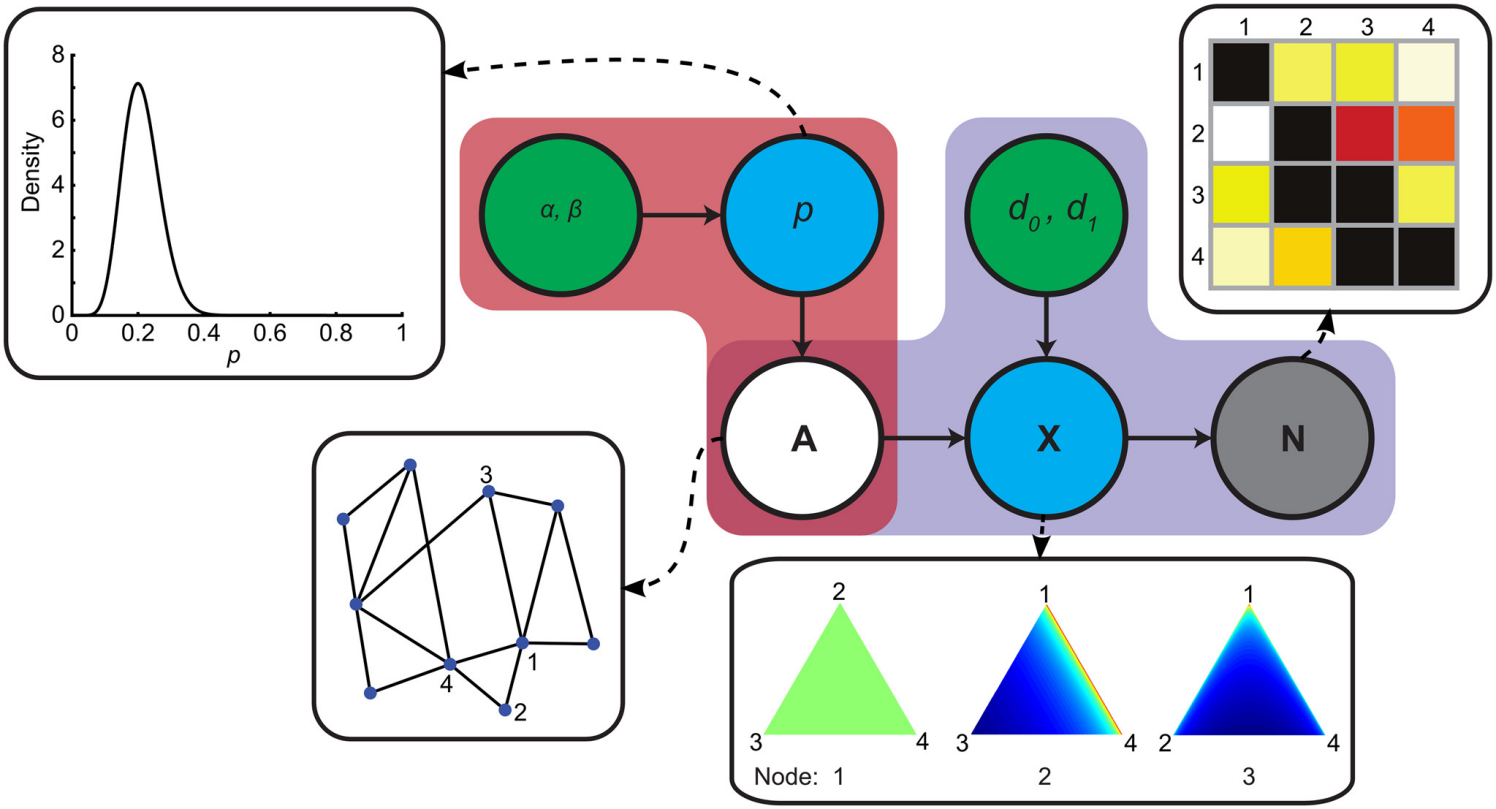
**Generative model of structural connectivity**. Red and purple regions indicate the prior and the likelihood function respectively. Green nodes indicate hyperparameters, blue nodes are latent variables, the gray node represents the streamline count matrix **N** and the white node is the unobserved variable of interest **A**. Insets show, from left to right, the prior distribution of *p*, a toy example of **A** and distributions over probability vectors in **X** for this toy graph. Distributions over probability vectors for three example nodes are plotted on a simplex. Each point on the simplex corresponds to a probability vector. The closer a point is to a vertex, the more mass is allocated to the corresponding element in the vector. The color gradients indicate the probability densities over probability vectors (red is high probability).

(1) P(N,A)=P(N∣A)P(A)

where *P*(**N** | **A**) denotes the likelihood function, modeling how an unobserved adjacency matrix leads to observed data, and *P*(**A**) denotes the prior on structural networks. Note that both the likelihood function and the prior depend on a set of hyper-parameters **θ**, which is left implicit in Equation (1). The generative model used in this paper is visualized in Figure 1.

### 2.1. Specification of the prior

The prior, indicated in red in Figure 1, models how whole-brain structural networks are generated. It allows incorporation of various assumptions about these networks that derive from background knowledge (Mukherjee and Speed, 2008). These assumptions can range from the very specific, like including known connections that are difficult to image, e.g., splenial fibers between ventral visual cortices (Dougherty et al., 2005), to the very abstract, such as incorporating the assumption that structural networks have small-world properties (Bassett and Bullmore, 2009; Bullmore and Bassett, 2011).

We wish to incorporate an assumption on graph density which can be modeled using a Beta-Binomial prior. We start by introducing a random variable *p* that represents the prior probability of the presence (*a*_*ij*_ = 1) or absence (*a*_*ij*_ = 0) of an edge between any pair of regions *i* and *j* in the adjacency matrix **A**. This edge existence is modeled using a Bernoulli distribution with parameter *p*, essentially implementing a weighted coin flip, where *p* is drawn from a Beta distribution. Hence:

$$\begin{array}{l} p|\alpha ,\beta \sim \text{Beta}(\alpha ,\beta )\\ \;a_{ij}|p\sim \text{Bernoulli}(p)\text{ for }i<j\text{ and }a_{ij}=a_{ji} \end{array}$$

where ~ denotes that the left-hand side random variable is distributed according to the density on the right-hand side. Values for α and β encode our prior knowledge on connection probability. Integrating out *p*, we obtain the following prior on structural networks:

(2) $$\begin{array}{l} P(A|\alpha ,\beta )=\int \text{Beta}(p|\alpha ,\beta )\left[\prod_{i\;<\;j}\text{Bernoulli}(a_{ij}|p)\right]dp\\ \;=\;\frac{B\left(e_{1}+\alpha ,e_{0}+\beta \right)}{B(\alpha ,\beta )} \end{array}$$

with $B(x,y)=\int_{0}^{1}t^{x\;-\;1}{\left(1-t\right)}^{y\;-\;1}dt$ the Beta function, *e*_1_ the number of edges in **A**, *e*_0_ the number of non-edges in **A**, and where we respect the constraints that **A** should be symmetrical and does not contain self-loops. In this prior, edges are coupled through their dependence on the global *p*. Hence, its distribution corresponds to a prior distribution on graph density. See the inset of Figure 1 for a plot of the prior on network density.

In our previous work, we used an Erdös-Rènyi model as the graph prior, which corresponds to a Bernoulli prior on the edges with a global *p*. In the beta-binomial prior, we place a hyper prior on *p* and integrate it out, which couples the edges through their dependence on *p*. The main benefit is that it allows us to express uncertainty in the expected graph density, hence it is a more flexible prior.

### 2.2. Specification of the likelihood function

In order to complete the model, we need to specify the likelihood function, indicated in purple in Figure 1, embodying a forward model that captures how structural networks lead to observed data. This raises the question of how to determine the presence of a connection, especially in the face of asymmetric streamline counts.

When modeling how structural networks lead to observed streamline counts, we assume that the presence of an edge between regions *i* and *j* increases the probability that a streamline is drawn from *i* to *j* (or vice versa) for 1 ≤ *j* ≤ *K* and *i* ≠ *j*. That is, for each region *i* we draw the probability vector **x**_*i*_ = (*x*_*i*1_, …, *x*_*i*(*K*−1)_) that sums to one and models the probability of streamlining from *i* to *j*, from the following Dirichlet distribution:

$$x_{i}|a_{i},d_{0},d_{1}\sim \text{Dirichlet}(d_{0}(1-a_{i})+d_{1}a_{i})$$

where **a**_*i*_ denotes the *i*-th row of the adjacency matrix **A**, excluding *a*_*ij*_. The hyper-parameter *d*_0_ influences the streamlining probability from region *i* to *j* in the absence of an edge between these regions. If an edge does exist between two regions, the streamlining probability is influenced by *d*_1_. For each region *i*, given a streamline probability vector **x**_*i*_, the vector of streamline counts **n**_*i*_ is drawn from a Multinomial distribution:

$$n_{i}|S_{i},x_{i}\sim \text{Multinomial}(S_{i},x_{i})$$

where *S*_*i*_ is the number of streamlines drawn by the tractography algorithm. The choice of the multinomial follows directly from the form of the data, i.e., counts over regions. The Dirichlet distribution is a conjugate prior for the multinomial and provides the link between the binary, symmetric graph and the asymmetric streamlining probabilities. In doing so, it allows us to distribute the probability mass in the streamline probability vector in a way that is consistent with both graph structure and streamline counts. Because we use a conjugate prior, we can integrate out the intermediate variable, **x**_*i*_, to obtain the following distribution on streamline count matrices:

$$P(N|A,d_{0},d_{1})=\prod_{i}\text{​}\left[\text{​​}\frac{S_{i}!}{\prod_{j}x_{ij}!}\frac{\Gamma \left(\text{​}\sum_{j}\alpha_{ij}\text{​}\right)}{\Gamma \left(\text{​}S_{i}+\text{​}\sum_{j}\alpha_{ij}\text{​}\right)}\prod_{j}\frac{\Gamma \left(x_{ij}+\alpha_{ij}\right)}{\Gamma \left(\alpha_{ij}\right)}\text{​​}\right]$$

where α_*ij*_ = *d*_0_(1 − *a*_*ij*_) + *d*_1_*a*_*ij*_ and the constants *S*_*i*_ are left implicit on the left-hand side.

This completes the specification of our generative model, the behavior of which is fully determined by the vector of hyper-parameters **θ** = (α, β, *d*_0_, *d*_1_). In order to develop an intuition for how the forward model leads to observed streamline counts, Figure 1 demonstrates, for a four-node network, how probability vectors are drawn and streamline counts are generated. With *d*_1_ = 1 and *d*_0_ < *d*_1_, probability vectors for node *i* are uniformly distributed over all target nodes *j* where *a*_*ij*_ = 1, that is, any probability vector is as likely as the next in the forward model. If *a*_*ij*_ = 0, i.e., there is no edge, the most likely vectors are those with low probability assigned to the unconnected node (note that the distribution remains uniform between the two connected nodes). Note that setting *d*_1_ > 1, encodes the assumption that streamlines from a region tend to be evenly distributed over connections, whereas *d*_1_ < 1 implies a tendency toward a “winner takes all” regime.

The model specification further demands *d*_0_ < *d*_1_, but setting an exact value for *d*_0_ can be difficult. The behavior of the model also depends on the number of connections a region has through the ratio between *d*_0_ and *d*_1_. This means *d*_0_ needs to be specified in accordance with assumptions on graph density as well as expected false positive rates. The relation between *d*_0_, node degree and false positive rate is illustrated in Figure S1. Simulations show that, with *d*_0_ and a node degree of 18 (connection density of 0.2), we expect 5% of the probability mass to be assigned to non-edges. This is equivalent to expecting a 5% false positive rate in the streamline counts.

### 2.3. Inference

The posterior distribution over structural networks cannot be calculated analytically, so we resort to a Markov Chain Monte Carlo (MCMC) approach to sample from this distribution (Gelman et al., 2003). We constructed a Metropolis sampler for our model which generates a proposal sample A^*^ from the previous sample **A**^*t* − 1^ by adding or removing a symmetric edge pair (*a*_*ij*_, *a*_*ji*_). The acceptance ratio for this proposal can be written as

(3) $$\begin{array}{l} \log\frac{P(A^{*}|N,\theta )}{P(A^{t\;-\;1}|N,\theta )}=\log\frac{P(N|A^{*},d_{0},d_{1})}{P(N|,A^{t\;-\;1},d_{0},d_{1})}\\ \;+\log\frac{P(A^{*}|\alpha ,\beta )}{P(A^{t\;-\;1}|\alpha ,\beta )}. \end{array}$$

The terms on the right hand side correspond to the likelihood function and the prior on networks respectively. A derivation for the likelihood term can be found in Hinne et al. (2013). The term representing the prior is obtained by plugging the result of Equation (2) into Equation (3). The set of samples generated by this inference procedure forms an approximation of the posterior over structural networks.

### 2.4. Graph-theoretical measures

**Figure 2:**
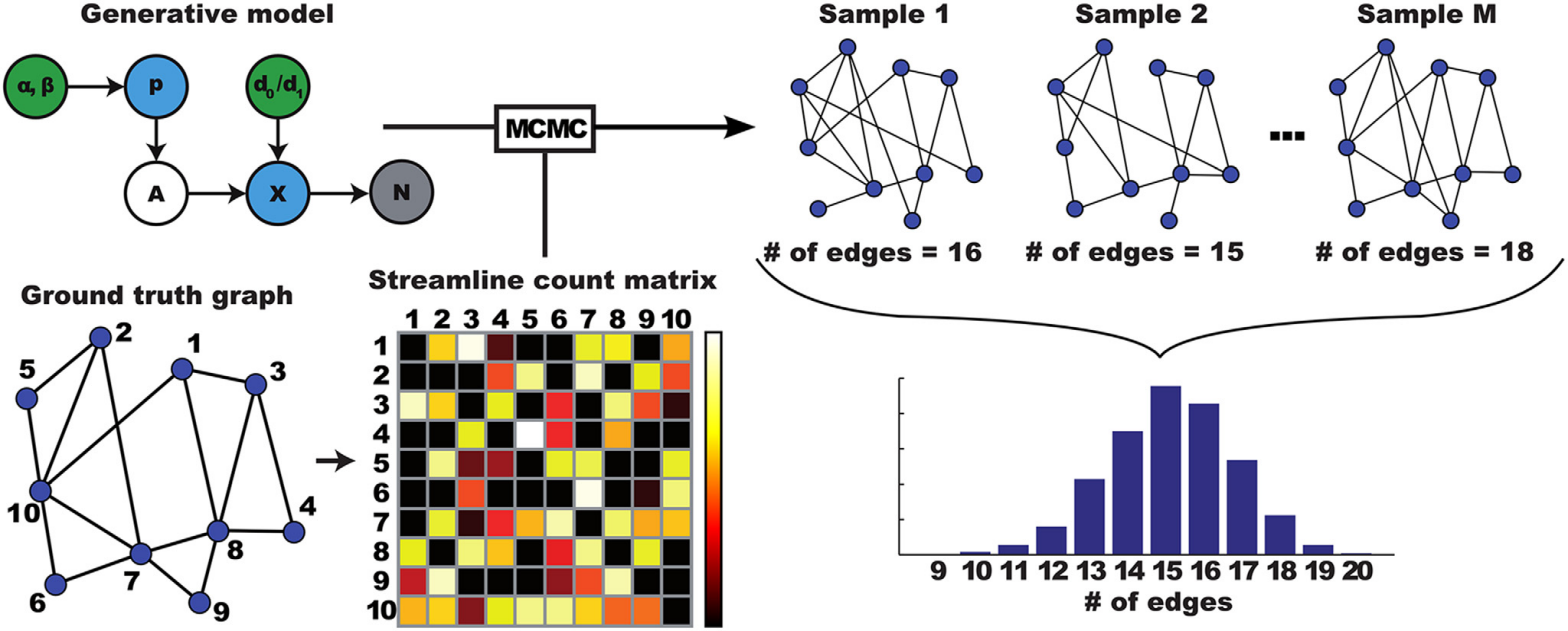
**Illustration of how a distribution over networks is translated to a distribution over graph properties**. A ground truth graph leads to streamlining data, which is used as input to an MCMC algorithm. This MCMC algorithm approximates inversion of the generative model given the data, by generating samples from the posterior distribution. Each of these sampled graphs can be analyzed using graph theoretical measures. Here, number of edges, resulting in a distribution over edge counts.

The posterior distribution over structural networks can be used to derive various quantities of interest as illustrated in Figure 2. The marginal probability of any given connection, for example, is given by the fraction of samples that contain this link. Likewise, the posterior over a graph measure is obtained by computing it for each individual sample from the posterior graph distribution. The result is a distribution of values for that particular graph measure.

In this paper, we consider a number of different graph measures. Small-worldness is a graph summary statistic that reflects the balance between local and global connectivity. High small-worldness has consistently been found in graphs derived from both functional and structural connectivity estimates (Bassett and Bullmore, 2009; Bullmore and Bassett, 2011) and has been linked to biologically relevant phenotypes (He et al., 2008, 2009; Schmitt et al., 2008; Yan et al., 2011). It is defined as the ratio between normalized estimates of the clustering coefficient and characteristic path length and is therefore influenced by both of these statistics. Modularity is a measure that reflects the presence of community structure in a graph, that is, it is a measure of well the graph can be partitioned into non-overlapping communities. In terms of brain networks, these communities represent functional subsystems of the nervous system. Node centrality is a measure of importance of a node in a network and there are numerous variants of centrality indices. One of the most straightforward examples is betweenness centrality, which is simply the fraction of shortest paths that pass through the node in question and can be used to identify hub regions in a network (Sporns et al., 2007). By computing these graph-theoretical measures for each of the MCMC samples, we obtain posterior distributions. Thus, uncertainty is propagated to the level of graph-theoretical analysis.

## 3. Results

In order to demonstrate the merits of Bayesian connectomics, we use diffusion imaging data collected for twenty subjects (these are shown in Figure S2 as streamline count matrices). Specifically, we show that we can produce graph summary statistics with an associated credible interval for individual subjects, which extends the interpretability of these statistics. To accomplish this, we estimated posteriors of network measures using the BaCon framework and compared these results with point estimates obtained from graphs based on thresholded streamline count matrices. The threshold for these was chosen such that thresholded network density was matched to the mean posterior network density for each subject individually. These graphs are visualized as adjacency matrices in Figure S3.

**Figure 3:**
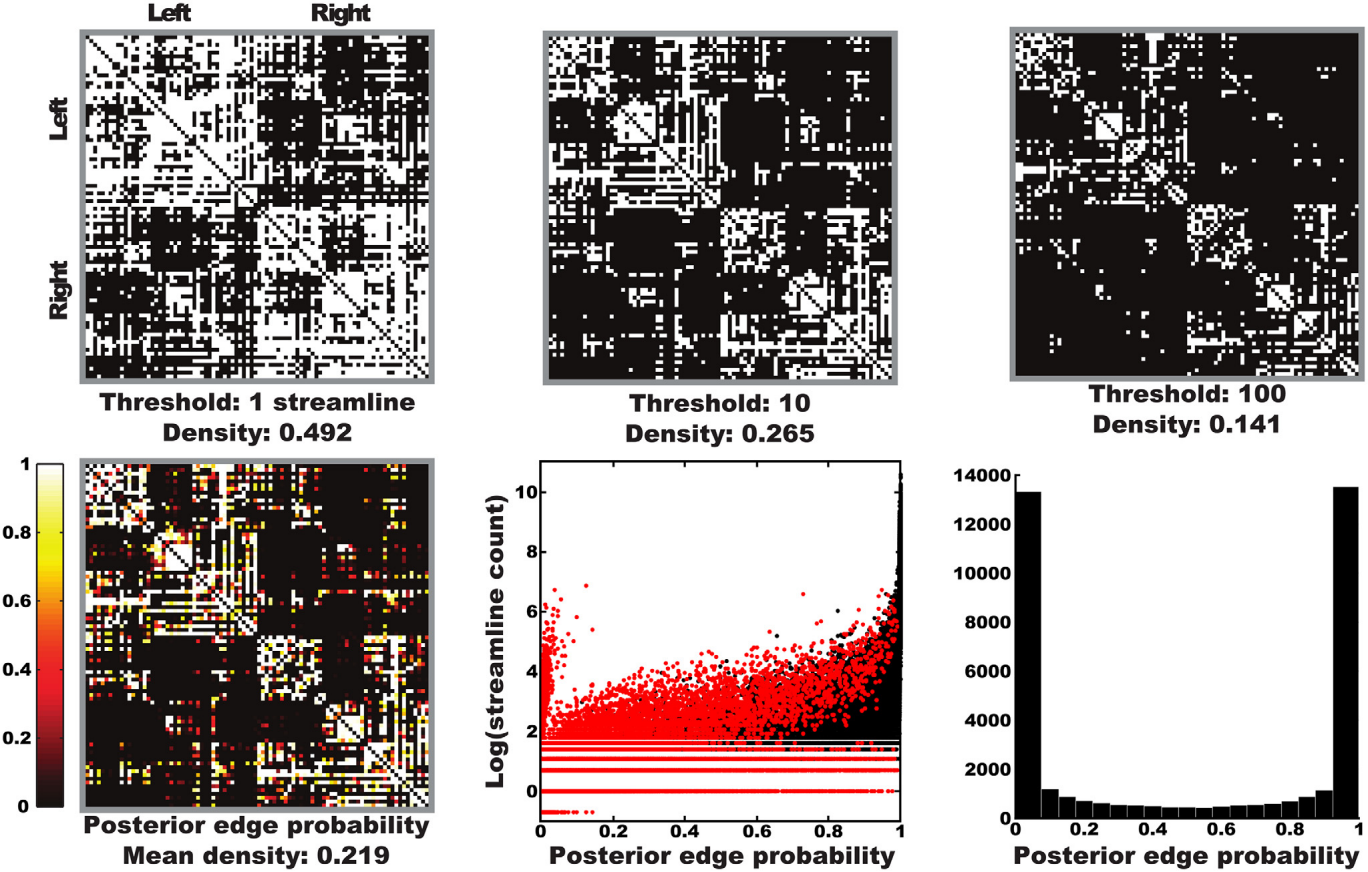
**Thresholded graphs and posterior edge probabilities**. Top row: illustration of the arbitrariness of thresholded graphs represented as adjacency matrices for a number of possible streamline thresholds for one subject (Subject 7). Bottom left: posterior edge probabilities for one subject as obtained with our Bayesian approach. Bottom middle: scatter plot of posterior edge probabilities vs. log streamline counts over all subjects, with edges present in both posterior and point estimates in black and those only in posterior in red (all edges in the point estimate were present in the posterior). Each edge is represented in both directions. The lower most line represents edges with zero streamline counts in one direction, these are the same edges as the “tuft” of high counts with low probabilities. Bottom right: histogram of non-zero edge probabilities over all subjects. The symmetric appearance is due to the mass of low probability edges being concentrated around very low probabilities.

The arbitrariness of thresholding is illustrated in the top row of Figure 3, where thresholded graphs are visualized as adjacency matrices for an example subject (Subject 8; the same subject will be used as example throughout this section). Clearly, the estimated structural graph heavily depends on the chosen threshold. The posterior edge probability matrix for that subject is also depicted in this Figure (Figure S4 for all subjects). Edge probabilities were obtained using our framework by calculating the ratio between samples containing a given edge and the total number of samples drawn. Plotting these edge probabilities against the streamline counts reveals that our approach is able to model strongly asymmetric streamlining distributions, although it does generally assign low edge probabilities probabilities to these edges. A histogram of non-zero edge probabilities reveals that our model is generally confident about either the presence or absence of edges, with relatively few intermediate probabilities.

**Figure 4:**
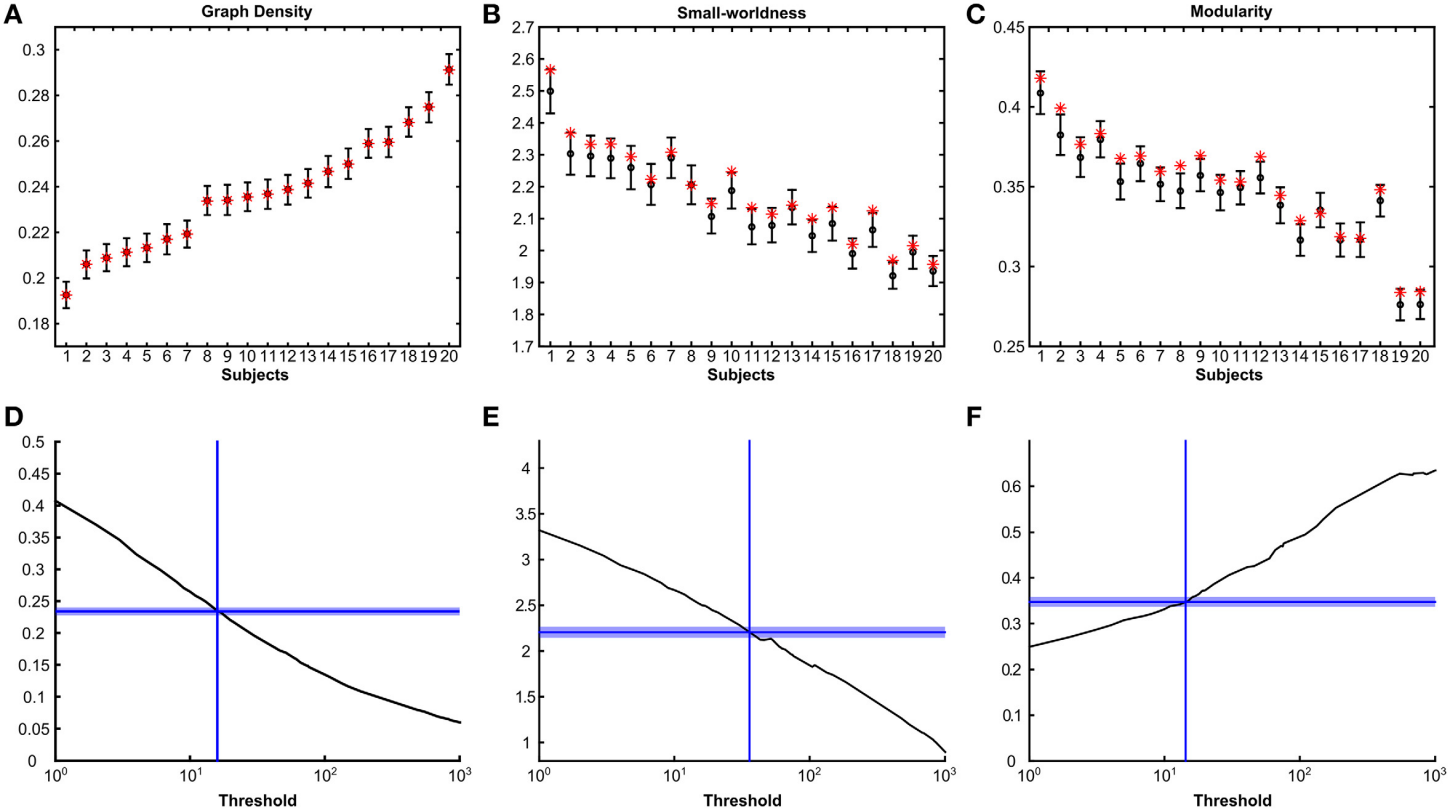
**Posterior densities and point estimates of graph measures**. Top panels show mean and 95% highest posterior density intervals (HPD) for graph density **(A)**, small-worldness **(B)** and modularity **(C)** for all subjects sorted by their mean posterior graph density. Point estimates for thresholded graphs are indicated with red stars. Bottom panels **(D–F)** show the same graph measures as a function of streamline threshold for one subject (subject 8). Only thresholds resulting in connected graphs are shown. Horizontal dashed lines indicate the mean posterior estimate and the shaded areas cover the 95% HPD interval.

The posterior distribution of networks also allowed us to subsequently generate estimates for graph-theoretical measures with credible intervals for individual subjects. Figures 4A–C show subject specific 95% highest posterior density (HPD) intervals (Murphy, 2012) for graph density, small-worldness and modularity, with subjects ordered with respect to posterior graph density. Subjects differed considerably in posterior graph density, most likely reflecting differences in data quality based on which inferences are made. Both small-worldness and modularity were highly dependent on graph density, as shown by the downward slope over subject in Figures 4B,C. Point and posterior estimates were in agreement for both small-worldness and modularity, with point estimates generally falling within the 95% HPD interval of the posteriors. However, point estimates were also systematically higher than posterior means, which implies that the networks obtained using thresholding are qualitatively different from those obtained using the Bayesian framework. In Figures 4D–F, the same measures are plotted as a function of threshold for an example subject (Figures S5–S7 show equivalent plots for all subjects). These figures show that posterior network densities correspond to an arbitrary range of thresholds. Moreover, posterior small-worldness and modularity densities were associated with a wider range of thresholds. These results indicate that these measures are not only affected by the choice of threshold but also has an associated uncertainty as captured by our framework.

**Figure 5:**
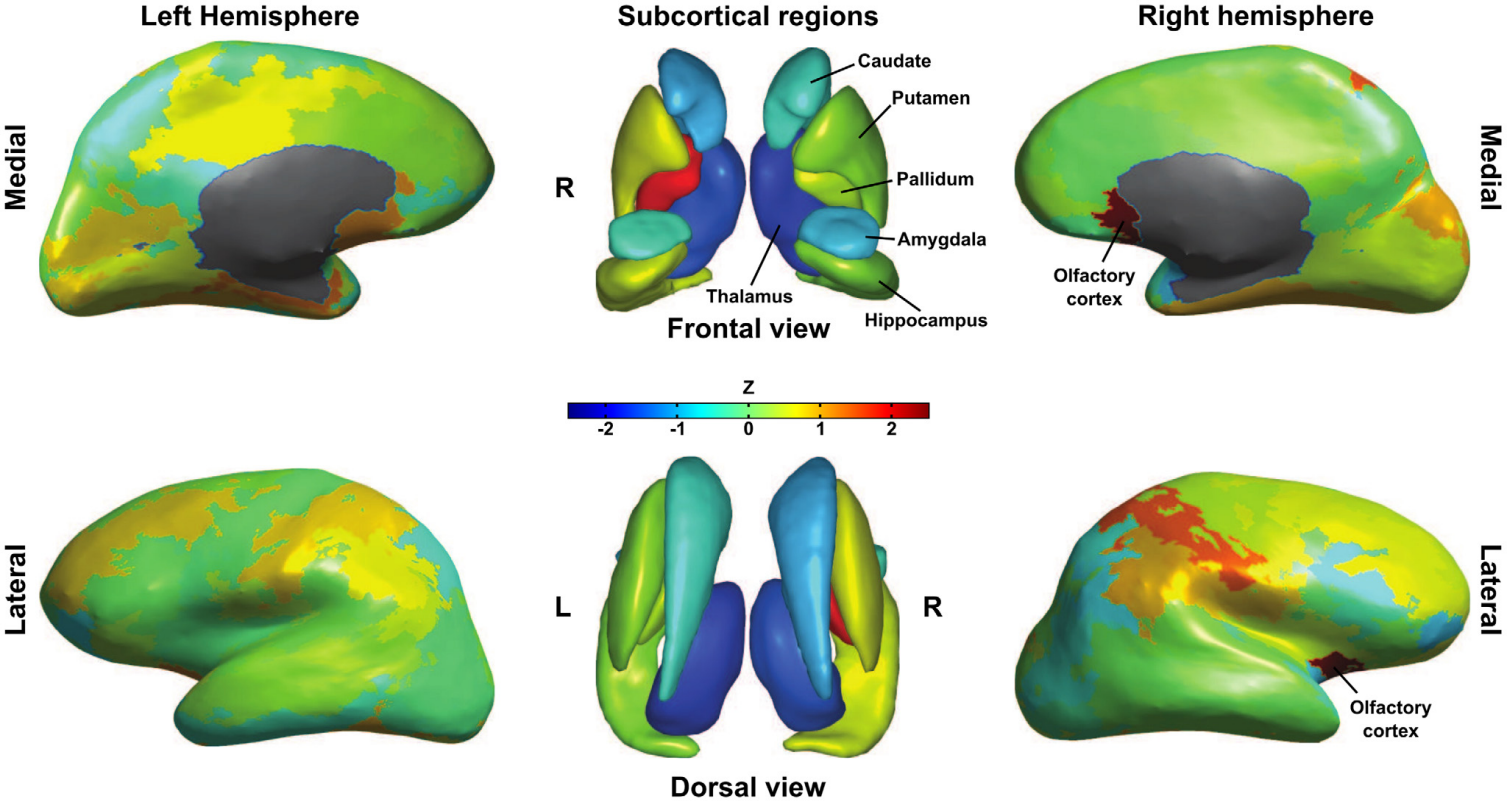
**Differences in betweenness centrality between point estimates obtained from thresholded graphs and posterior distributions**. For each subject we computed the distance between point and posterior estimates as their difference normalized to the posterior standard deviation. Subcortical and inflated cortical surfaces show the median of this distance over subjects. Note the consistent difference for right olfactory cortex, left pallidum and both thalami.

In the remainder of this section, we examine estimates obtained for region-specific graph-theoretical measures. Node centrality is a measure of importance of a node in a network and there are numerous variants of centrality indices. One of the most straightforward examples is betweenness centrality, which is simply the fraction of shortest paths that pass through the node in question. Betweenness centrality can be used to identify hub regions in a network (Sporns et al., 2007). To visualize the relationship between point and posterior estimates of betweenness centrality, we computed a distance *z*_*i*_ as follows for every region *i*: *z*_*i*_ = (μ^*t*^_*i*_ − μ_*i*_)/σ_*i*_, where μ^*t*^_*i*_ is the point estimate of betweenness centrality, μ_*i*_ the median posterior betweenness centrality and σ_*i*_ the standard deviation of this posterior. The median of *z*_*i*_ across subjects is plotted on the (sub)cortical surfaces in Figure 5.

For most nodes, there was no consistent difference between point and posterior estimates of betweenness centrality. Notable exceptions to this, however, are the left and right thalamus, which had consistently lower betweenness centrality in the point estimates as compared to posterior means. Another interesting difference can be found in the left pallidum, which was consistently higher in point estimates. Thresholded graphs also returned a higher betweenness centrality for right olfactory cortex as compared with the posterior distribution. To help explain these differences, surrogate group-level connection probabilities were estimated by averaging thresholded graphs as well as the posterior probability matrices over subjects. For all non-zero probabilities in either approach, we took the difference between thresholding and posterior estimates and *Z*-transformed these differences. Looking at the difference between the average connectivity profiles over subjects, we find that thresholding returns lower connection probabilities (*Z*<−2.3) between left thalamus and bilateral cingulate cortex, ipsilateral insula, temporal pole and amygdala as well as contralateral superior frontal cortex and paracentral lobule. Right thalamus had lower connection probabilities to ipsilateral orbito-frontal, cingulate and inferior parietal cortices as well as ipsilateral amygdala. A lack of these connections could very well explain the lower thalamic centrality in thresholded graphs and may indicate that thresholding misses out on relevant connections. Thresholded graphs had higher global connectivity probabilities for right pallidum, but these could not be isolated to any specific connection. Surprisingly, right olfactory cortex showed lower connection probabilities (*Z*<−2.3) with ipsilateral amygdala and contralateral olfactory, anterior cingulate, medial superior frontal and rectus cortex in thresholded graphs. This suggests that the difference in betweenness-centrality is due to more distant edge configurations.

## 4. Discussion

The Bayesian connectomics framework presented in this paper illustrates that one can obtain structural network estimates, as well as the uncertainty thereof, using a generative approach which relies on Bayesian inference. As shown in Figure 3, thresholding at a streamline count of one results in implausibly dense graphs, begging the question what threshold is optimal. Our framework, however, does not require an arbitrary threshold. Instead, it estimates both the number and configuration of connections supported by the data under our model.

Since our framework produces a distribution of networks, this can be used to formulate connection probabilities with a clear interpretability that is not afforded by thresholding. It should be noted that these connection probabilities are marginal probabilities that integrate out their dependence on the rest of the graph. In general, as shown, the distribution of networks can be subjected to any graph-theoretical analysis, providing posterior estimates for these measures with credible intervals. The general agreement between measures obtained from thresholded graphs and our model shows that both approaches uncover similar topological features. That being said, the employed threshold was informed by the posterior graph density estimate. Setting a threshold is considerably less straightforward without such information.

Providing estimates of uncertainty enables a meaningful comparison of graph measures for individual subjects, or even regions within individual subjects for node-wise metrics, where such a comparison was previously only interpretable at the group level. Such estimates of uncertainty can become especially important when correlating graph-theoretical measures with phenotypes such as intelligence (Li et al., 2009), motor skills (Wang et al., 2013) or development (Meunier et al., 2009). In a clinical setting, the Bayesian framework may lead to more sensitive markers for disorders which have been shown to be related to differences in structural connectivity, e.g., schizophrenia (Fornito et al., 2012) or Alzheimer's disease (Lo et al., 2010; Reijmer et al., 2013; Tijms et al., 2013). These approaches rely on reproducibility within subjects, which was not assessed in this work and should be addressed in future research.

Ultimately, the quality of structural estimates obtained using the Bayesian approach depends on both the quality of the data and the validity of the employed model. In terms of data quality, one important factor is the choice of parcellation, especially when examining graph metrics. The size and number of parcels greatly influence graph metrics (de Reus and van den Heuvel, 2013) and the same holds for our posterior estimates. In this paper, we used a parcellation of 90 cortical and sub-cortical regions, which is a rather small number of regions. However, streamlines could be aggregated at more fine-grained levels to form the data matrix, though at the cost of increased computational time.

From the modeling point of view, different functional forms of the prior and likelihood function, reflecting different assumptions, may further improve the inferences drawn by the model presented in this paper. In contrast to thresholding or deterministic approaches, such assumptions are easily incorporated in the framework. In this paper, we used a prior on global graph density reflecting our knowledge that local density can vary within a graph, while assuming very little otherwise. Other knowledge about structural networks can easily be integrated within the prior, for instance, that these networks tend to have small-world properties (Bassett and Bullmore, 2009; Bullmore and Bassett, 2011). Moreover, there are connections that are known from, for example, macaque studies, which are difficult to establish using diffusion MRI (Dougherty et al., 2005)^1^. Both of these types of information, i.e., global and local, can be incorporated into a prior on structural networks. Exponential random graph models (ERGMs) are another way to generate a prior distribution of binary networks and encode prior assumptions on graph structure (Robins et al., 2007), although their usage is computationally more costly. Next to the development of more suitable priors, research may focus on extending the forward model. For example, although in the current model we set *d*_0_ and *d*_1_ to the same value for all subjects, these Dirichlet parameters could also be sampled by placing a hyper-prior on them, allowing adaptation to individual subjects' data.

A number of studies employ weighted graphs (e.g., Lo et al., 2010; Raj et al., 2012) and one shortcoming of the model proposed here is that it can only produce a posterior over binary graphs. Weighted graphs would be a more realistic representation of brain networks as not all connections are created equal. It should be possible to generate distributions of weighted networks by formulating a sensible forward model that links a matrix of connection strengths to the appropriate data. This formulation is not straightforward, however, due to interpretation issues inherent to DWI (Jones et al., 2013).

Summarizing, the BaCon framework presents a principled approach to estimating brain networks as well as graph-theoretical measures thereof. Using a generative model easily allows incorporation of new model assumptions into the existing framework. In general, we advocate the use of a hypothetico-deductive approach in which developed models are continuously adjusted in the light of incoming data (Gelman and Shalizi, 2013). It is our hope that the presented framework as well as its possible extensions will improve the interpretability of results obtained from connectivity analysis studies.

## 5. Methods

### 5.1. Data acquisition

Data consisted of T1 and DWI images for twenty subjects, which was a subset of the data used in van Oort et al. (2014) and kindly provided by the authors. The reader is referred to this publication for details on the acquisition protocol. In short, T1 images were obtained with 1 mm^3^ isotropic resolution and DWI data with 256 diffusion directions and 2 mm^3^ isotropic voxels.

### 5.2. Preprocessing

All preprocessing was performed using FSL 5.0 (http://fsl.fmrib.ox.ac.uk). Structural scans were segmented using FAST (Zhang et al., 2001) and FIRST (Patenaude et al., 2011). The preprocessing steps for the diffusion data were conducted using FSL FDT (Behrens et al., 2003) and consisted of correction for eddy currents and estimation of the diffusion parameters. We used FDT Probtrackx 2.0 (Behrens et al., 2003, 2007) for probabilistic streamlining, where gray matter voxels served as targets while voxels on the gray-white matter boundary served as seeds. The target mask was a combination of the gray matter mask output from FAST and the interior voxels of the subcortical segmentation from FIRST, this mask also served as termination mask. The seed mask consisted of the mixeltype mask representing the mixture of gray and white matter voxels from FAST combined with the boundary mask for the subcortical structures from FIRST. Overlap between seed and target mask was removed from the seed mask.

For each of the seed voxels 5000 streamlines were drawn, with a maximum length of 2000 steps (step size was 0.5 mm). Streamlines were stopped when they reached a voxel in the target mask. Streamlines were restricted by fractional anisotropy and they were discarded if a sharp angle (<80°) occurred or their length was less than 2 mm. The output thus obtained is a matrix **N**, where *n*_*ij*_ is the number of streamlines drawn from voxel *i* in the seed mask to voxel *j* in the target mask. This matrix was collapsed into 90 cortical and subcortical areas as defined by the AAL atlas (excluding cerebellum). Streamlines were summed over all voxels per region, resulting in a 90 × 90 aggregated connectivity matrix which ranges over regions rather than voxels. The aggregated streamline count matrices are freely available through www.ccnlab.net.

### 5.3. Network estimation

Structural networks were estimated from probabilistic streamline count data using either a thresholding approach or using our Bayesian connectomics approach. In order to compare results, subject-specific thresholds were chosen such as to include the same number of edges as were present on average in the posterior networks for a given subject (edges were randomly selected in case of a tie). The streamline count matrices were symmetrized prior to thresholding by summing counts from either direction (*i* to *j* and vice versa).

Hyper-parameters of the generative model were chosen as follows. We chose α = 14 and β = 53 as a vague prior on sparse networks. The forward model has parameters *d*_0_ and *d*_1_, these parameters control the distribution of probability vectors for a given network. Note that a probability vector is drawn for each row in the adjacency matrix separately. With *d*_1_ > 1, one would encode the expectation that, for any given region, there is uniformity in streamlining probabilities and hence, given the large number of streamlines drawn, uniformity in streamline counts over connections with other regions. On the other end, setting *d*_1_ < 1 encodes the expectation that a single connection will be responsible for generating most streamlines from a given region. We set *d*_1_ = 1 and in doing so, encode that we are agnostic to the way streamlining probabilities are distributed over connections. Setting *d*_0_ < *d*_1_ encodes the assumption that false positives should occur less frequently than true positives. We simulated draws from a dirichlet distribution with varying *d*_0_ and connection density, while keeping *d*_1_ = 1. From these simulations, we obtained the proportion of probability mass assigned to non-edges, which corresponds with the expected false positive rate. We selected *d*_0_ = 0.01 so that the expected false positive rate at a connection density of 0.2 was approximately 5%.

For the initialization of our MCMC sampler, in order to minimize burn in time, we used a thresholded, symmetrized streamline count matrix with a density based on the mode of the prior on graph density. We stored one sample for each complete graph update, i.e., after all unique edges were flipped in random order. Posterior network distributions were approximated for each subject by drawing 5000 samples in two parallel chains, for a total of 10000 samples.

Brain network measures were derived from thresholded networks and sampled networks using native Matlab scripts and the Matlab BGL package (http://dgleich.github.com/matlab-bgl). For each uniqe graph density, 100 random, density-matched graphs were generated to normalize clustering and path length. Small-worldness was computed as the ratio of these normalized estimates. Modularity was selecting the highest score from 100 runs of a Louvain modularity optimization algorithm for each posterior graph sample and graph point estimate.

## Author contributions

Marcel A. J. van Gerven, Max Hinne, Tom Heskes, Ronald J. Janssen designed the analysis. Ronald J. Janssen and Max Hinne performed preprocessing and data analysis. Marcel A. J. van Gerven, Max Hinne, Tom Heskes, and Ronald J. Janssen wrote the manuscript.

### Conflict of interest statement

The associate editors Prof. Bielza and Prof. Larrañaga declares that, despite having coauthored a paper with the author Dr. Heskes, the review process was handled objectively. The authors declare that the research was conducted in the absence of any commercial or financial relationships that could be construed as a potential conflict of interest.

## Footnotes

^1^Or, more rarely, large streamline counts where connections are known not to exist.
