# Supplementary material for: Quantifying uncertainty in brain network measures using Bayesian connectomics
Source: Front Comput Neurosci. 2014 Oct 8;8:126. doi: 10.3389/fncom.2014.00126 (PMC4189434; doi:10.3389/fncom.2014.00126)
Supplement: Supplementary file 1 [file DataSheet1.PDF]

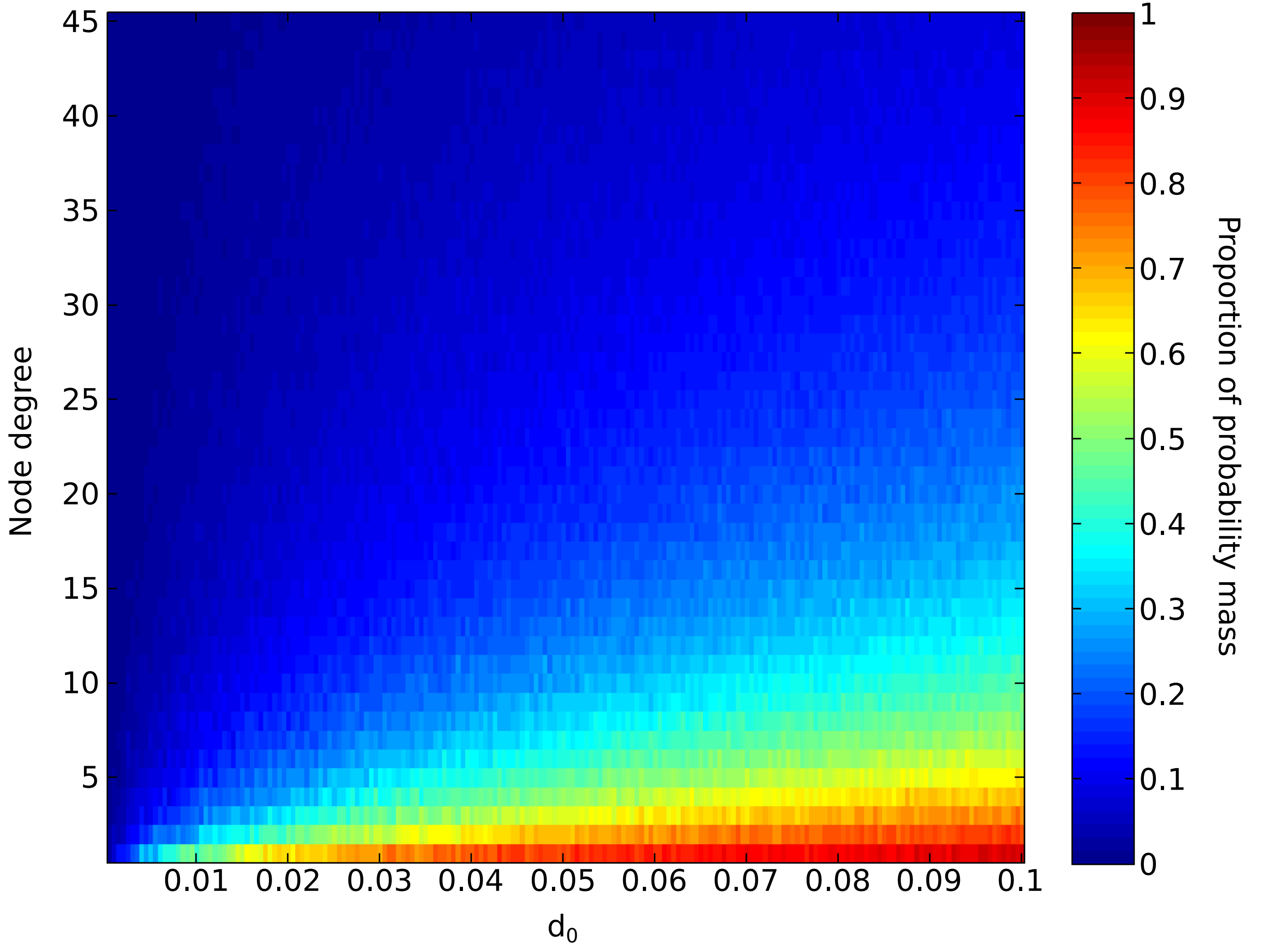

Supplemental Figure 1: Expected proportion of probability mass assigned to non-edges with d_1 set to 1. The abscissa and ordinate represent d_0 and node degree respectively whereas the color axis indicates the expected proportion of total probability mass assigned to non-edges as a whole. This can loosely be interpreted as the expected false positive rate. As d_0 increases, so does the expected false positive rate. With higher degree, there is a decrease in false positive rates, as there is more opportunity for mass to be assigned to true positives.
