# Supplementary material for: Quantifying uncertainty in brain network measures using Bayesian connectomics
Source: Front Comput Neurosci. 2014 Oct 8;8:126. doi: 10.3389/fncom.2014.00126 (PMC4189434; doi:10.3389/fncom.2014.00126)
Supplement: Supplementary file 2 [file DataSheet2.PDF]

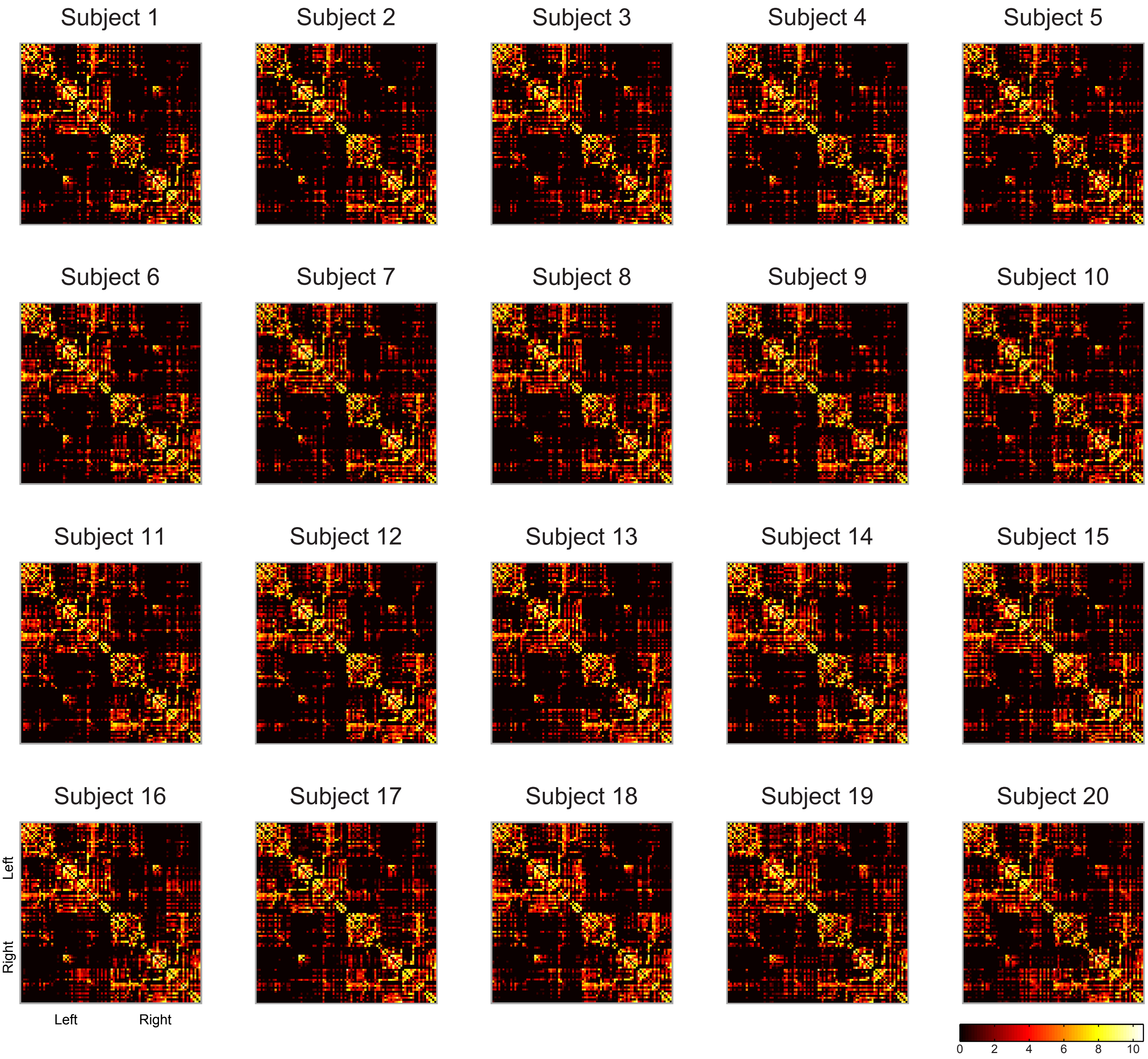

Supplemental Figure 2: Streamlining matrices for all subjects. Matrix elements represent the log transformed streamline counts.
