# Supplementary material for: Quantifying uncertainty in brain network measures using Bayesian connectomics
Source: Front Comput Neurosci. 2014 Oct 8;8:126. doi: 10.3389/fncom.2014.00126 (PMC4189434; doi:10.3389/fncom.2014.00126)
Supplement: Supplementary file 3 [file DataSheet3.PDF]

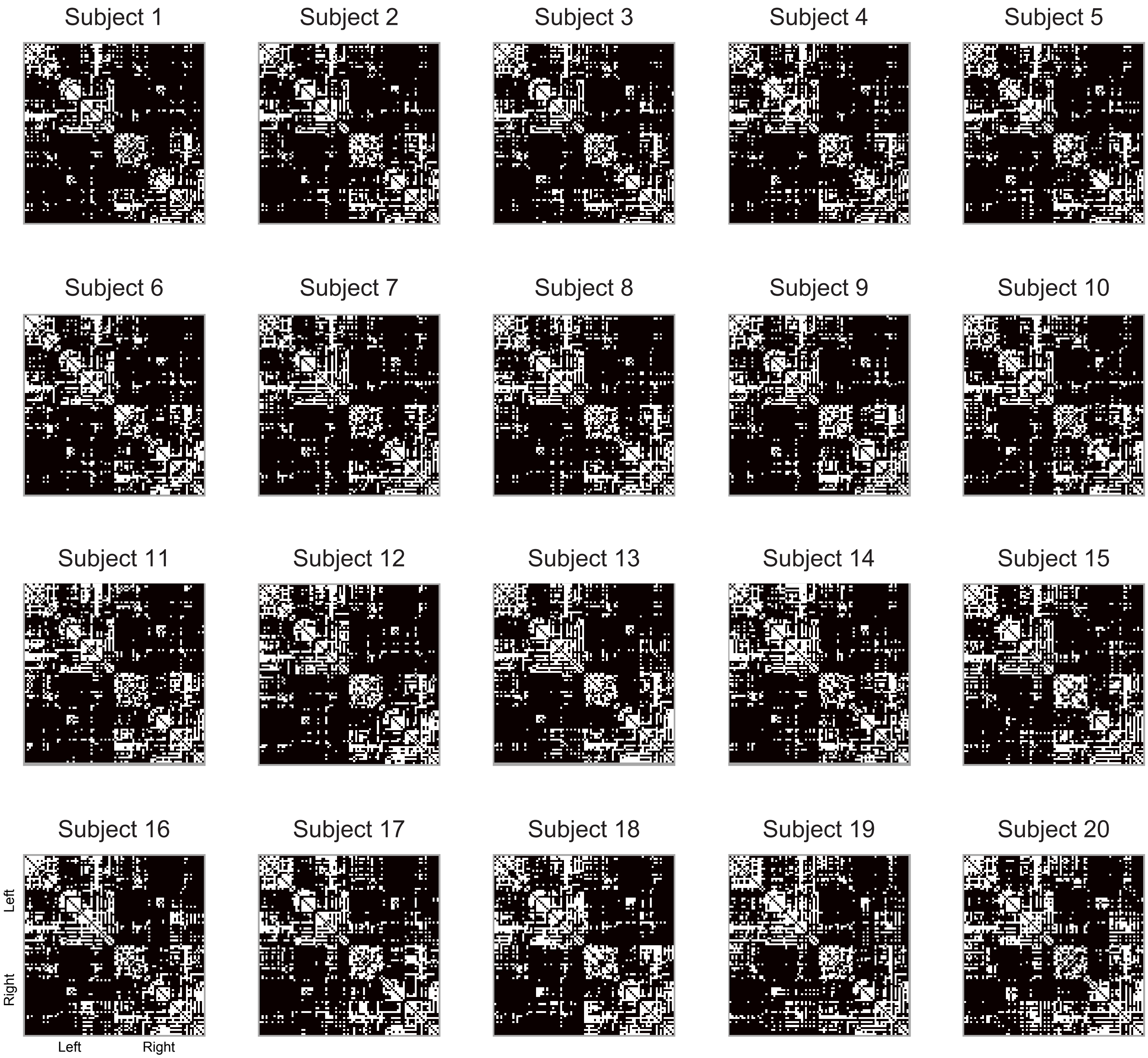

Supplemental Figure 2: Thresholded graphs for all subjects. Graphs were thresholded to match the subject's mean posterior graph density.
