# Supplementary material for: Quantifying uncertainty in brain network measures using Bayesian connectomics
Source: Front Comput Neurosci. 2014 Oct 8;8:126. doi: 10.3389/fncom.2014.00126 (PMC4189434; doi:10.3389/fncom.2014.00126)
Supplement: Supplementary file 4 [file DataSheet4.PDF]

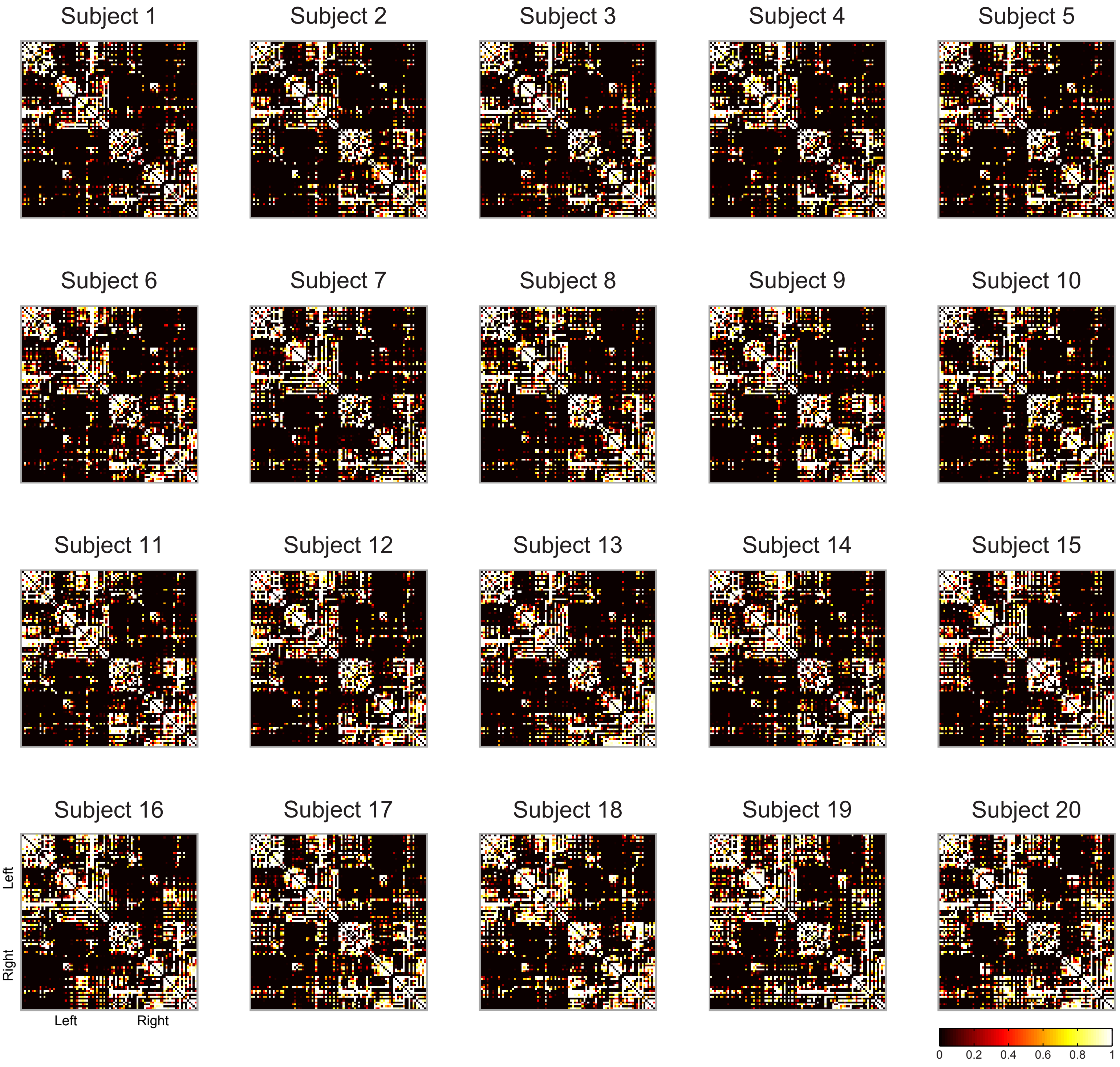

Supplemental Figure 4: Posterior edge probability matrices for all subjects. Matrix elements are edge probabilities obtained by taking the ratio of the number of samples containing that edge to the total number of samples drawn.
