# Supplementary material for: Quantifying uncertainty in brain network measures using Bayesian connectomics
Source: Front Comput Neurosci. 2014 Oct 8;8:126. doi: 10.3389/fncom.2014.00126 (PMC4189434; doi:10.3389/fncom.2014.00126)
Supplement: Supplementary file 6 [file DataSheet6.PDF]

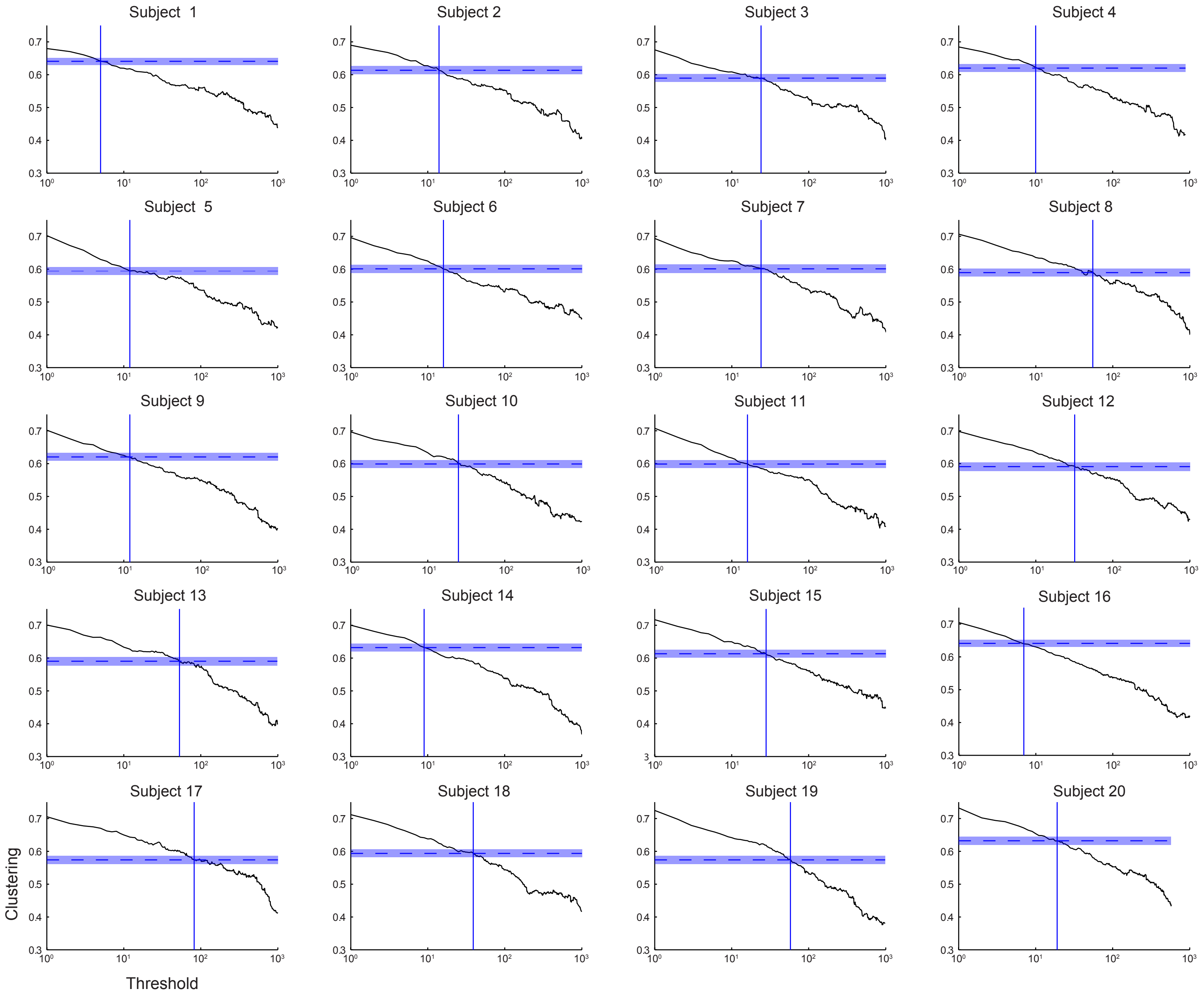

Supplemental Figure 6: Clustering as a function of streamlining threshold for all subjects. Horizontal dashed lines indicate the mean posterior clustering and vertical lines indicate thresholds with the closest correspondence in clustering. Shaded areas cover the 95% highest posterior density. Note the log scale on the abscissa and the considerable variation in corresponding thresholds.
