# Supplementary material for: Quantifying uncertainty in brain network measures using Bayesian connectomics
Source: Front Comput Neurosci. 2014 Oct 8;8:126. doi: 10.3389/fncom.2014.00126 (PMC4189434; doi:10.3389/fncom.2014.00126)
Supplement: Supplementary file 7 [file DataSheet7.PDF]

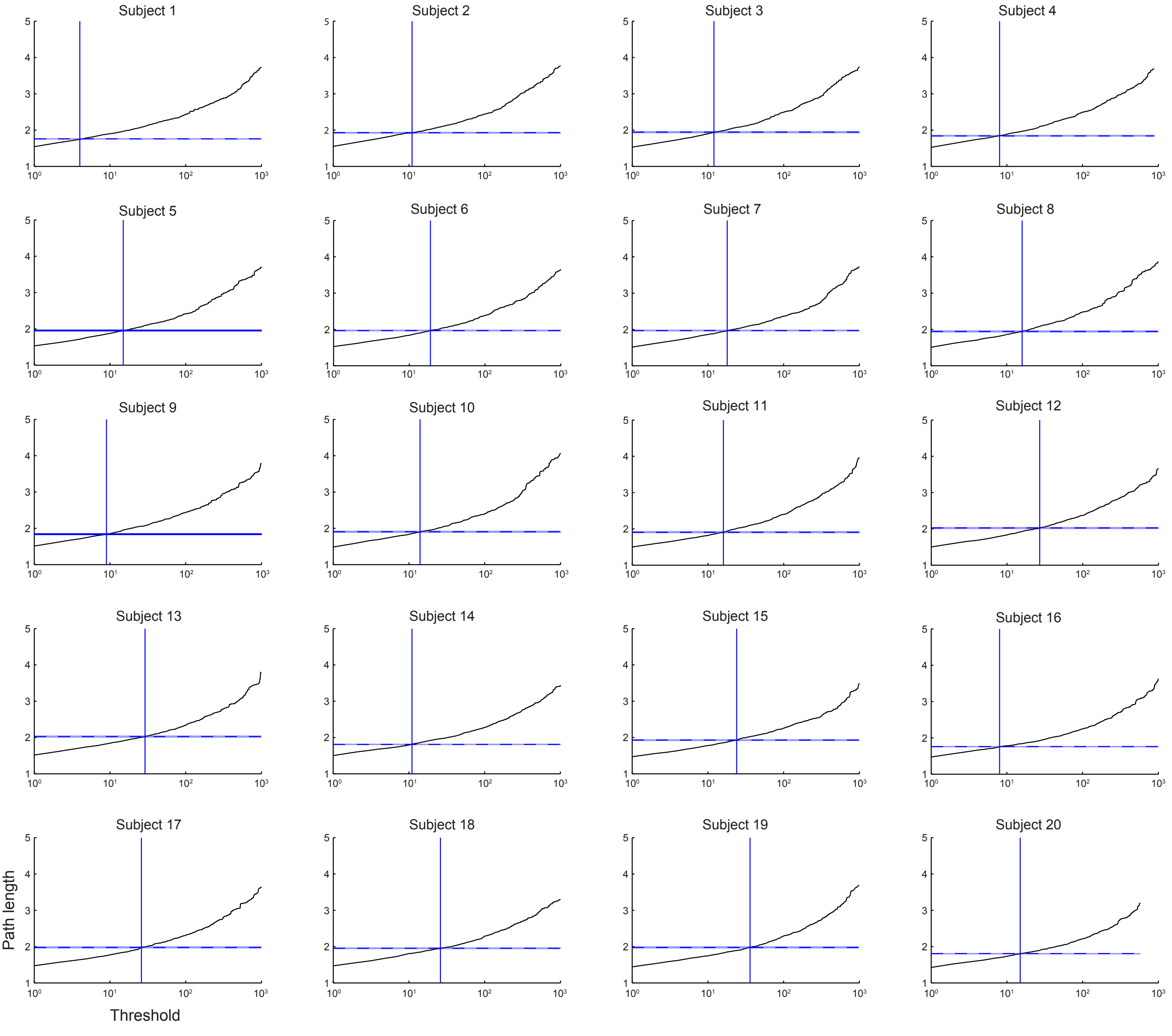

Supplemental Figure 7: Path length as a function of streamlining threshold for all subjects. Horizontal dashed lines indicate the mean posterior path length and vertical lines indicate the threshold with the closest correspondence in path length. Shaded areas cover the 95% highest posterior density. Note the log scale on the abscissa.
